# Myelomatous Pleural Effusion: A Rare Occurrence in Multiple Myeloma

**DOI:** 10.7759/cureus.26045

**Published:** 2022-06-17

**Authors:** Mohammad Asim Amjad, Zamara Hamid, Srinivasarao Ramakrishna, Renee Frank, Pius Ochieng

**Affiliations:** 1 Internal Medicine, The Wright Center for Graduate Medical Education, Scranton, USA; 2 Internal Medicine, Shifa International Hospital, Islamabad, PAK; 3 Pulmonary and Critical Care, Geisinger Commonwealth School of Medicine, Scranton, USA; 4 Anatomic and Clinical Pathology, Geisinger Community Medical Center, Scranton, USA; 5 Pulmonary and Critical Care, Geisinger Community Medical Center, Scranton, USA

**Keywords:** immunoglobulin kappa light chains, rare cause of pleural effusion, myelomatous pleural effusion, multiple myeloma, pleural effusion

## Abstract

Various factors can cause pleural effusion in multiple myeloma patients. Myelomatous pleural effusion (MPE) is an uncommon but potentially life-threatening complication of multiple myeloma with a poor prognosis. After ruling out all other probable causes, the present case reports MPE in a patient with IgG kappa multiple myeloma.

## Introduction

Multiple myeloma is defined by the malignant proliferation of plasma cells, resulting in increased monoclonal protein synthesis, an aberrant immunoglobulin (Ig) produced by neoplastic plasma cells, and reduced or normal concentrations of normal Ig [1,2]. While most clinical symptoms such as lethargy, bone pain, and infections are associated with marrow infiltration, specific unexpected presentations have been reported such as pleural effusion or infiltrates in the lungs, bone lesions, and plasmacytomas in the thorax. Pleural involvement is uncommon in multiple myeloma, occurring in 6% to 14% of cases owing to non-myeloma-related reasons and 1% due to multiple myeloma-related causes [3-5]. The following case illustrates a pleural effusion caused directly by multiple myeloma known as myelomatous pleural effusion (MPE).

## Case presentation

**Figure 1:**
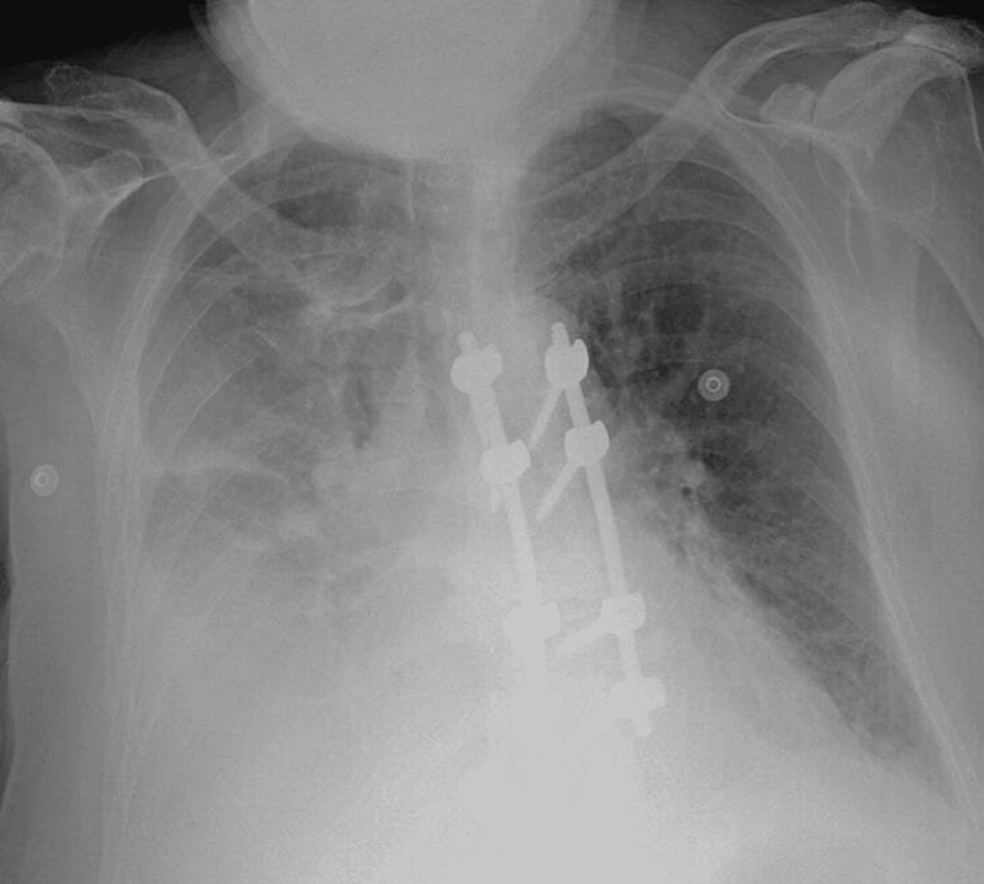
X-ray chest indicating a pleural effusion on the right side. The figure was generated entirely for this publication and gained agreement from the patient to post it.

**Figure 2:**
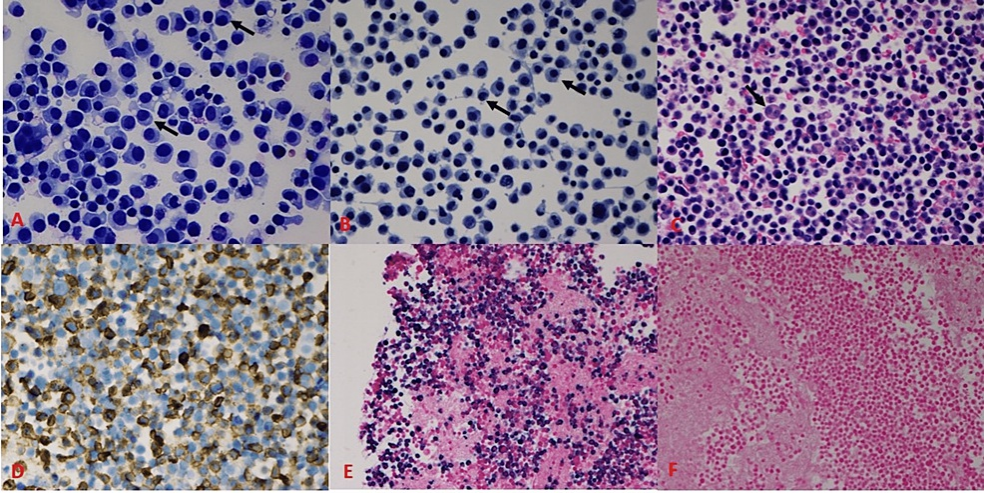
Pleural fluid cytopathology showing numerous plasmacytoid cells. A and B: Cytospin preparations of abundant plasmacytoid cells with perinuclear hofs (arrows) and coarse nuclear chromatin pattern (arrows) (400X DiffQuik stain and Papanicolaou stain, respectively). C: Cellblock preparation of plasmacytoid cells, rare mesothelial cells are noted in the background (arrows) (400X Hematoxylin and eosin stain). D: Immunohistochemical stain CD138 highlighting plasma cells (400X). E and F: In situ hybridization stain for immunoglobulin light chains showing kappa restriction (200X Kappa and Lambda, respectively).

**Figure 3:**
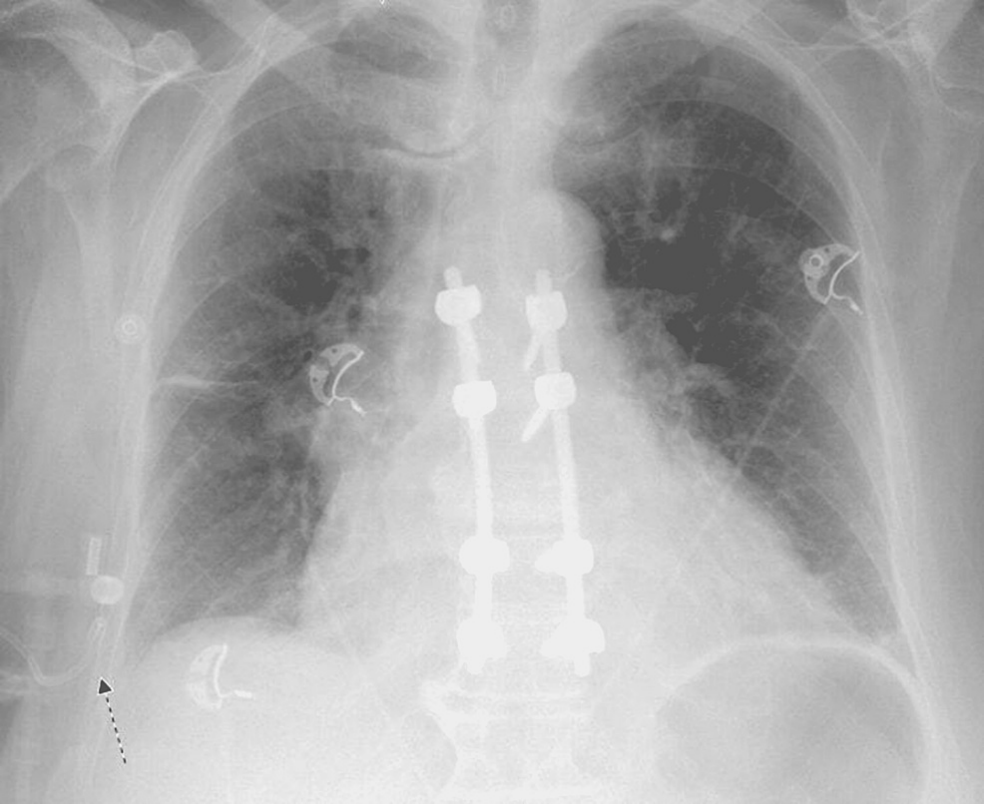
X-ray of the chest after insertion of the pleural catheter (arrow). The figure was generated entirely for this publication and gained agreement from the patient to post it.

An 86-year-old female with advanced (IIIa) IgG‐Kappa multiple myeloma, diagnosed four years earlier, presented with exertional dyspnea for one month. Six months prior, she had a pathological fracture of the T9 vertebrae producing spinal cord compression, which was treated with an emergency laminectomy and pedicle screw stabilization of the spine. While undergoing autologous stem cell transplantation evaluation, chemotherapy was initiated with bortezomib, cyclophosphamide, and dexamethasone. The patient developed dyspnea on exertion after two cycles of induction chemotherapy, limiting her exercise tolerance to a few steps. She denied any additional symptoms that could point to a cardiac cause. She had no history of smoking, vaping, recent travel, sick contacts, occupational exposure, or pets at home. She had a temperature of 100.1°F, a heart rate of 90 beats per minute, a respiratory rate of 22 breaths per minute, a blood pressure of 130/80 mmHg, and an oxygen saturation of 95% in room air. There was less air intake on the right side and percussion dullness on the chest exam. The neurological, gastrointestinal, and cardiovascular exams found nothing abnormal. The results of the general laboratory workup including complete blood count and a comprehensive metabolic profile (Table 1) were unremarkable. Hypogammaglobulinemia on serum electrophoresis, and monoclonal kappa light chains on serum immune electrophoresis. An X-ray (Figure 1) and computed tomography of the chest revealed a new moderate to large right pleural effusion. Thoracocentesis yielded an exudative effusion that was lymphocytic predominant (Table 2). The results of bacterial and mycobacterial cultures were negative. Pleural fluid cytopathology (Figure 2) showed abundant, monotonous, enlarged cells on cytospin preparations. The enlarged cells had eccentrically placed nuclei with condensed chromatin and occasional perinuclear hofs were present in the cellular cytoplasm. In the background of the plasmacytoid cells were rare mesothelial cells. Immunohistochemical stain CD138 was performed on the cell block preparation and revealed the abundant monotonous cells were plasma cells. In situ hybridization studies for kappa and lambda immunoglobulin light chains indicated the plasma cells were kappa restricted. Due to recurring pleural effusions, she had therapeutic thoracocentesis followed by a pleural catheter placement (Figure 3). The patient died after nearly a month of septicemia despite multiple treatment regimens.

**Table 1:** General laboratory work up

Test Name	Patient Values	Reference Range	Units
WBC	10.2	4.0-10.80	K/uL
RBC	3.97	4.00-5.25	M/uL
Hemoglobin	11.4	14.0-16.8	g/dL
Hematocrit	35.1	40.0-48.4	%
RDW	17.5	11.5-15.5	%
MCV	88.4	82.0-99.5	fL
MCH	28.7	27.0-34.0	Pg
MCHC	32.5	32.0-36.0	g/dL
Platelet Count	107	140-400	K/uL
Monocytes	3.9	1.0-11	%
Neutrophils	61.0	40.0-75.0	%
Lymphocytes	32.4	18.0-42.0	%
Eosinophils	0.04	0.0-6.0	%
Absolute Basophils	0.04	0.0-0.2	K/uL
Absolute Eosinophils	0.04	0.0-0.7	K/uL
Absolute Lymphocytes	3.25	1.0-4.8	K/uL
Absolute Monocytes	0.39	0.3-1.0	K/uL
Absolute Neutrophils	6.11	1.8-7.8	K/uL
C-reactive protein	1	<3.0	mg/dl
Erythrocyte Sedimentation Rate	25	0-35	mm/h
Urea	22	15-39	mg/dl
Creatinine	1.0	0.57-1.11	mg/dl
Sodium	137	135-146	mEq/L
Potassium	5.1	3.5-5.1	mEq/L
Phosphorus	3.3	2.5-4.9	mg/dl
Total Bilirubin	0.32	0.2-1.0	mg/dl
Lactate Dehydrogenase	220	84-246	UI/L
Alanine Aminotransferase	37	30-65	UI/L
Aspartate Aminotransferase	20	15-37	UI/L
Albumin	2.7	3.4-5.0	g/dl
Free T4	1.36	0.76-1.46	ng/dL
TSH	0.596	0.358-3.740	UI/mL
Folic Acid	14	3.1-17.5	ng/dL

**Table 2:** Pleural Fluid Analysis Analysis revealed exudative effusion with lymphocytic predominance.

Test Name	Result	Reference Range	Units
Clarity, Fluid	Cloudy	Straw	
Protein	2.8	1-2	g/dl
Glucose	69	< 60	mg/dl
Total Nucleated Cell Count	10,824	< 3000	cells/ul
Neutrophils	5	0-1	%
Lymphocytes	85	18-36	%
Monocytes	10	64-80	%
RBC	2000	< 0	cells/mL

## Discussion

Multiple myeloma (MM), a plasma cell cancer, is responsible for 10% of blood malignancies [6]. Plasma cells cause M protein hypersecretion, a defective immunoglobulin that causes the well-known CRAB syndrome, including hypercalcemia, renal failure, anemia, and bone lytic lesions [1]. Even though these malignant cells are most frequently located in the bone marrow, they can also be found in extramedullary tissues. The thorax is a well-known site for skeletal disease (lytic bone lesion or pathological fracture), plasmacytomas, pulmonary infiltrates (including infections), and pleural effusions, amongst many other findings [4,6].

Most pleural effusions in MM are benign (non-myelomatous), occurring in 6-14% of cases [7,8]. Multiple causes contribute to its development, including congestive heart failure, pulmonary embolism, renal failure with or without nephrotic syndrome, and amyloidosis [3,8]. It occurs in less than 1% of cases where pleural effusion is directly attributable to MM, known as myelomatous pleural effusion (MPE) [5]. Rodriguez et al. were the first to identify MPE as a diagnosis, exhibiting monoclonal protein and plasma cells in the pleural fluid and histological confirmation with pleural biopsy in 1994 [8]. Later on, Cho et al. conducted the most extensive analysis to date, reviewing 734 cases with MM and discovering that 7% (54) of the cases had pleural effusion, with 2.3% (19) classified with MPE [9]. Due to IgA MM's proclivity for invading extraosseous structures, the bulk of IgA MM's proclivity for invading extraosseous structures, the bulk of cases in the literature have been associated with it, making our case an outlier. They emerge late in the disease and carry a poor prognosis [10].

The disease's pathogenesis has been attributed to a number of variables, the most prevalent of which include tumor invasion of the pleura (referred to as hematogenous dissemination), concomitant skeletal or lung plasmacytomas, and lymphatic blockage due to the involvement of mediastinal lymph nodes. Invasive therapy (surgery) and bone fractures are also known to enhance the risk of MPE in MM patients as they contribute to increasing the extramural spread of the disease [3,11,12]. Another case in point was the pleural effusion that developed in our patient following spine surgery several months prior to her presentation and for which no other explanation could be found. Furthermore, Natori et al. discovered several chromosomal abnormalities, including a translocation affecting the immunoglobin heavy chain (Ig H) area on chromosome 14q32, which could be the disease's starting point [13]. Genetic changes are important to understand because they can be linked to clinical prognosis. 

MPE requires diagnostic thoracentesis, including protein analysis, fluid cytology tests, and pleural biopsy. Despite the existence of specific diagnostic criteria, diagnosing MPE is difficult. Pleural cytology is a rapid, sensitive, and effective method for finding and detecting phenotypically abnormal cancer cells with a 60% accuracy rate and can allow for early detection [14-16]. Pleural biopsy is a risky blind operation that is less appealing as a diagnostic tool since MPE affects the pleural in an uneven pattern. As a result, it was not pursued in our patient. In addition, flow cytometry of pleural fluid is a good adjunct tool to the usual strategy of cytology for the improved diagnosis of MPE [15].

MPE does not have a specific treatment, although systemic chemotherapy in conjunction with radiotherapy is occasionally used as the mainstay of treatment in certain circumstances, with pleurodesis serving as palliative care. None of the previously known cases survived more than four months from the onset of their effusion, with the median survival duration being three months, which is similar to our case, where our patient died after only one month from complications [17,18].

## Conclusions

To summarize, pleural effusion is a complicated condition that MM patients can induce for various reasons. Diagnosing MPE demands a detailed investigation due to the therapeutic and prognosis consequences. Additional clinical studies are necessary for the future to discover the appropriate treatment strategy with more precision, as they are resistant to treatment and frequently relapse despite current options.
